# Characterization and functionality of two members of the SPFH protein superfamily, prohibitin 1 and 2 in *Leishmania major*

**DOI:** 10.1186/s13071-018-3195-8

**Published:** 2018-12-04

**Authors:** Teresa Cruz-Bustos, Ana Karina Ibarrola-Vannucci, Isabel Díaz-Lozano, José Luis Ramírez, A. Osuna

**Affiliations:** 1 Department of Parasitology, Faculty of Science, University of Granada, Granada, Spain; 2 Molecular Parasitology Research Group (CTS-183), Institute of Biotechnology, University of Granada, Granada, Spain; 3 Instituto de Estudios Avanzados, (IDEA), Caracas, Venezuela; 4 Instituto de Biología Experimental, Universidad Central de Venezuela, Caracas, Venezuela; 5 Karolinska Institute, Stockholm, Sweden

**Keywords:** *Leishmania major*, Prohibitin 1, Prohibitin 2, Oxidative stress, Immunochemistry, Functionality

## Abstract

**Background:**

Leishmaniasis, a disease caused by parasites of the genus *Leishmania*, infects roughly 12 million people worldwide, with about two million new cases per year. Prohibitins (PHBs) are highly conserved proteins belonging to the stomatin-prohibitin flotillin-HflC/K (SPFH) protein superfamily. In this study, we examine the potential functions of two proteins of *Leishmania major*, PHB1 and PHB2, as well as how they might help protect the protozoan against oxidative stress.

**Results:**

By immunolocalization in the parasite cells, PHB1 appeared in the mitochondria and plasma membrane, whereas PHB2 was grouped in the nucleus. When *Leishmania* cells were under oxidative stress, PHB1 migrates towards the plasma membrane and the paraxial rod, while PHB2 remained in the nucleus and near the kinetoplast. PHB1 presented higher mRNA levels than PHB2 in the amastigotes and the infective metacyclic forms. The mRNA expression of both prohibitins was affected by the presence of the Fe^3+^ ion. PHBs inhibited the Fenton reaction, where reactive oxygen species could nick DNA, implying that they play a crucial role in controlling oxidative stress.

**Conclusions:**

Here, we propose that PHBs may help to protect membranes and DNA against superoxide ions, thus enhancing the survival capacity of the protozoan by controlling the ROS within the phagosome of the macrophages where the parasite multiplies.

## Background

All the members of the genus *Leishmania* are intracellular protozoa that live and multiply in the macrophages of their vertebrate hosts, causing a number of different pathological manifestations known as leishmaniasis. Approximately 12 million people are affected by this disease worldwide, with about two million new cases per year [1–3].

Some *Leishmania* species cause chronic disorders known as cutaneous, mucocutaneous or diffuse cutaneous leishmaniasis, in which lesions appear on the skin and mucous membranes. In another form of the disease, visceral leishmaniasis, the parasites live as amastigote forms inside the macrophages of the skin, spleen, liver or bone marrow [4]. In the present study, we investigate the characteristics and function of prohibitins 1 and 2 (PHB1, PHB2) of *Leishmania major*, the etiological agent of cutaneous leishmaniasis in the Old World.

Prohibitins (PHBs) are highly conserved proteins belonging to the stomatin-prohibitin flotillin-HflC/K (SPFH) protein superfamily, which have the common PHB domain. The members of this superfamily can be found in all eukaryotic cells, bacteria and archaea [5], and they are usually anchored to cell membranes in eukaryotic cells, including mitochondrial membranes [6].

Prohibitins in particular have been located in the inner membrane of the mitochondria, exerting various mitochondrial functions and stabilizing the mitochondrial membrane by acting as chaperones for other proteins [7]. The first function attributed to mammal prohibitin was to inhibit cell division in tumours [8]. Currently, this inhibitory effect is not considered to be due to the protein itself, but rather to its interaction with transcription factor E2F and with retinoblastoma (Rb) proteins (E2F suppressors) [5].

In human cells, prohibitins are key to cell-signalling pathways and have been associated with congenital pathologies related to innate immunity and iron homeostasis [9, 10]. In previous studies in yeast [11] PHB complexes have been shown to participate in the respiratory chain as chaperone/holdase proteins.

Prohibitins have been described and studied in the Kinetoplastida protozoan *Trypanosoma brucei* [12] and *Leishmania donovani* [13]. The inhibition of the PHB expression in *T. brucei* affects its mitochondria integrity by failing to stabilize *de novo* synthesis of other mitochondrial-associated proteins, thus causing a loss of permeability and an increased sensitivity to apoptosis [12].

In *L. donovani*, Jain et al. [13] discovered a positive relationship between a higher expression of PHB1 on the parasite’s surface and greater infectivity. Recently PHB1 has been proposed as a potential antigen for vaccination against *L. infantum* [14].

Here, we describe the location and some potential functions of *L. major* PHB1 and PHB2, as well as how these proteins might help protect the protozoan against oxidative stress (ROS) when the Fe^3+^ ion is linked to them. These proteins appear to participate in the intracellular survival of the protozoan inside the phagosome of the vertebrate macrophages where the intracellular amastigote forms develop. This is the first description of PHB2 in a *Leishmania* species, and, in agreement with Dias et al. [14] regarding *L. infantum* prohibitins, we propose that the blockage of the PHB1 and PHB2 function could open new strategies for treatment or vaccination against this global disease.

## Methods

### Culture methods

Isolates of *L. major*-Friedlin [MHOM/IL/81/Friedlin, (clone VI)] (Leishm) *L. panamensis*-(MOHM/PA/71/LS94) (Leishp), *L. braziliensis* LQ2 (MHOM/PE/95/LQ-2 (Leishb) and *L. amazonensis*-(MHOM/BR/77/LTB0016) (Leisha) strains were grown at 25 °C in RPMI medium containing 10% heat-inactivated foetal bovine serum (ICFS) at pH 7.2.

When the parasite culture reached the exponential phase, the flagellates were collected by centrifugation (1000× *g* at 4 °C) for 10 min and then sub-cultivated. The pellets (10 × 10^6^ flagellates) were washed three times in phosphate-buffered saline (PBS), pH 7.2, centrifuged, and then stored at -80 °C until use.

### PCR and cloning

*Leishmania* genomic DNA was extracted from the pellet removed after centrifugation of the parasite culture using the DNeasy Blood and Tissue kit (Qiagen, Hilden, Germany) following the manufacturer’s protocol. The purified DNA was precipitated with isopropyl alcohol, dried, resuspended in Milli-Q water and stored at -20 °C until use.

PCR was carried out with the DNA samples at concentrations between 25 and 100 ng, 10 pMol of the primers, 2.0 mM MgCl_2_ (Dominion), 20 mM for each of the dNTPs (Dominion), 2 μl of buffer 10× (25 mM Tris-HCl pH 9, 50 mM KCl), and for a total of 20 μl of the mixture 1 U of Taq Polymerase (Dominion).

For PHB1 (XP_001682244.1) the primers used were PHB1-F (5'-AAG GAT CCA TGT CGA AGT TGC TGC AGA AGG TTG CCA TC-3') and PHB1-R (5'-TAA AAA GCT TTC ACC TCG ACA TGT TCA TCA TCA GCA TGT TCG-3'). For PHB2 (XP_003722404.1), the primers used were PHB2-F (5'-AGG GAT CCA TGG CGG CCG AGG CGC GGA AGA AGA TGA A-3') and PHB2-R (5'- TAA AAA GCT TTT ACT TCG TCC CGG AAT GAT CGA-3').

PCR was performed in a Thermocycler C-1000 connected to CFX96 for Real-Time (Bio-Rad, Hercules, California USA); the amplification program having a heat denaturation cycle at 95 °C for 3 min, 30 cycles (at 94 °C for 1 min, at 65 °C for 1 min, at 72 °C for 1 min), and a final extension at 72 °C for 8 min. The PCR product size was confirmed by agarose gel electrophoresis and then purified using a kit (Qiagen) following the instruction manual QI Aquick PCR. The bands were sequenced with a BigDye Terminator v1.1 cycle sequencing kit (Applied Biosystems, Carlsbad, California, USA).

After verification of the correct cloning in pGEM-T easy (Promega, Columbia, South Carolina, USA) and confirming PHB1 and PHB2 reading frames, positive plasmids were used to transform *E. coli* JM109. The inserts were then sub-cloned in the expression vector pQE-30Xa vector (Qiagen). Bacteria hosting the recombinant plasmids were grown until an OD_600_ 0.6 was reached, and the expression of the recombinant proteins was induced by adding IPTG (1 mM), followed by incubation for 3 h. The culture was then centrifuged at 4000× *g* for 20 min. The supernatant was stored at -80 °C and the pellet re-suspended in lysis buffer A (Na_2_HPO_4_ 20 mM; 500 mM NaCl; 10 mM EDTA; 5 mM β-mercaptoethanol; 0.35 mg/ml lysozyme, pH 7.4) and incubated at room temperature (RT) for 30 min before being sonicated and centrifuged at 10,000× *g* for 30 min. The pellet was once again treated with lysis buffer B (Na_2_HPO_4_20 mM; Urea 8 M; NaCl 0.5 M and Imidazol 5 mM, pH 8) under constant stirring for 1 h until it solubilized; it was then centrifuged at 10,000× *g* for 20 min. Both the supernatant and the pellet were analysed by SDS-PAGE. Recombinant proteins were purified using a Ni-Sepharose column (His GraviTrap, GE Healthcare, Chicago, Illinois USA) following the manufacturer’s instructions.

### Immunoserum production and antibody purification

The antisera against the recombinant *Leishmania* prohibitins were obtained in mice after intramuscular injection with 50 μg of Leish r PHB1 and in rabbits with 500 μg of Leish r PHB2 with complete Freund’s adjuvant. This first injection was followed by three more injections applied every 10 days with the same antigens plus incomplete Freund’s adjuvant. The animals were exsanguinated two weeks after the final injection. The sera were checked by ELISA in multi-well plates coated with 10 μg/well of each recombinant PHB in 0.1 M carbonate/bicarbonate coating buffer (pH 8.6). The plates were blocked with 2% skimmed milk (Molico, Nestlé) in PBS (pH 7.2) containing 0.05% Tween 20 (Merck KGaA, Darmstadt, Germany). Sera with titres above 1:800 were pooled and stored at -80 °C until use.

The IgGs from the two immunosera were purified by affinity chromatography using a Protein G HP Spin Trap column (GE Healthcare), and then the specific antibodies were obtained by immunoabsorption on PVDF strips containing the purified recombinant proteins. The specific IgG antibodies were recovered from the strip with 0.1 M glycine buffer at pH 4.5; the pH was restored immediately to neutrality by adding 1 M Tris-HCl buffer, pH 8.0, supplemented with 0.1% purified bovine albumin and stored at -80 °C.

### SDS-PAGE and western blot analysis

To test the quality and specificity of the sera produced against the PHB recombinant proteins, we loaded 20 μg of protein from different purification samples and mixed with 2× Laemmli sample buffer [15]. Samples were loaded on 12.5% PGE-SDS gels and afterwards the electrophoresis gels were stained with Coomassie blue or silver nitrate [16]. Protein bands were transferred onto a PVDF membrane (Mini ProBlott Membranes, Applied Biosystems, Foster City, CA, USA) using a Bio-Rad transblot. Membrane strips were then incubated for 2 h at 37 °C with diluted 1:50 sera and, after several washes with PBST (PBS + Tween20), they were incubated for 2 h at RT with rabbit anti-mouse IgG (whole molecule)-peroxidase antibody (Sigma Aldrich, St. Louis, Missouri, USA) for Leish r PHB1 or with goat anti-rabbit IgG (whole molecule)-peroxidase antibody (Sigma Aldrich) at 1:1,000 for Leish r PHB2. Samples were developed using diaminobenzidine tetrahydrochloride (Sigma Aldrich) as a substrate (0.05% w/v) and H_2_O_2_ (dilution 1/5000).

### Protein sequencing

The protein bands that in the western blot were recognized by the pool of positive sera were retrieved from the gels, sequenced and identified at the “Servicio de Proteómica del Centro de Biología Molecular Severo Ochoa (CBMSO)” in Madrid, Spain. The bands were excised manually and digested automatically *in situ* with a robot digester (Bruker, Billerica, Massachusetts, USA) using trypsin following a previously described protocol [17]. The digestion products (containing the peptides) were acidified with trifluoroacetic acid (0.1% final concentration), dried in a Speed Vac (Thermo Fisher Scientific, Waltham, Massachusetts, USA ) and finally resuspended in TA (0.1% trifluoroacetic acid, 33% acetonitrile). A 0.5 ml aliquot was placed on an anchor-chip plate (Bruker), using 2.5-dihydroxybenzoic acid (DHB) as a matrix, at a concentration of 5 g/l *via* the “fast evaporation” method. The samples on the plate were analysed using an Autoflex matrix assisted laser desorption ionization time-of-flight (MALDI-TOF) mass spectrometer (Bruker) equipped with a reflector. The mass spectra from these experiments were used as a peptide fingerprint to identify proteins using Mascot and Profound databases.

### DNA and protein sequence analysis

The nucleotides and amino-acid sequences were BLAST searched in the TriTryp database (http://tritrypdb.org/tritrypdb/). Sequences of *L major* PHB1 (LmjF16.1610) and PHB2 (LmjF35.0070) were aligned with prohibitins from different species using the Clustal Omega software.

The structural motifs were searched for via the proteomic tool server of Expasy (https://www.expasy.org/) using the following programs: GOR IV [18], ProtScale [19], TopPrep [20] [21], BigPI Predictor [21–23], SignalP [24], Motifscan [25], Swissmodel [26], Myristolator [27], CSS-Palm [28], NetPhos 2.0 [29], SulfoSite [30] and InterPro.

### cDNA synthesis from total RNA samples and real-time PCR (RT-PCR)

The mRNA expression for PHB1 and PHB2 was quantified by quantitative RT-PCR (qRT-PCR). Additionally, mRNA was quantified for *L. major* (Friendlin strain), promastigotes, metacyclic promastigotes and amastigotes, as well as for the promastigote forms of *L. amazonensis* M2903, *L. braziliensis* LQ2 and *L. panamensis*.

Total mRNA was isolated from the different *Leishmania* forms using RNeasy and the Oligotex mRNA kit (Qiagen). For reverse transcription, an iScript cDNA synthesis kit (Bio-Rad) was used and quantitative RT-PCR (qRT-PCR) was performed in a final volume of 10 μl per reaction using a CFX96 Touch™ Real-Time PCR Detection System (Bio-Rad). Briefly, 1 μl of sample DNA (50 ng/μl) was added to 5 μl of a master mix Ssofast Evagreen Supermix (Bio-Rad) and nuclease-free water with primers PHB1-qF (5'-GCA CCT TCG GTC TCG ACT AC-3') and PHB1-qR (5'-GGA TGT CTA CCA GCG AGA GG-3') for prohibitin 1 and PHB2-qF (5'-CGC GGA AGA AGA TGA ACG-3') and PHB2-qR (5'-CGG GCA CAA AGA AGA CTG AC-3') for prohibitin 2, all adjusted to a final concentration of 300 nM. Polymerase activation was carried out at 95 °C for 2 min. The PCR program included 39 cycles of denaturation at 95 °C for 10 s, annealing and extension at 60 °C for 20 s. SYBR Green fluorescent emission was measured at the end of the elongation step. Subsequently, a melting-curve program was applied with a continuous fluorescent measurement starting at 65 °C and ending at 95 °C (ramping rate of 0.1 °C/s).

The 18S small subunit ribosomal RNA (18S SSU rRNA) from *Leishmania* (constitutive gene) was used to normalize the amount of sample analysed as previously described [31, 32]. Primers for 18S LEI-18S 1 (5'-ACT CAC GGC CTC TAG GAA TGA-3') and LEI-18S 2 (5'-TCG ATC TCC ACA CTT TGG TTC T-3') and primers for LEI-18S 1 (5'-ACT CAC GGC CTC TAG GAA TGA -3') and LEI-18S 2 (5'-TCG ATC TCC ACA CTT TGG TTC T-3') were used to amplify 152 bp fragments.

The samples were quantified according to the ΔC_T_ method, in which *18S*/Leish PHB1 or Leish PHB2 ratio = 2^(CT 18S - CT Leish PHB1 or Leish PHB2)^. All assays were made in triplicate.

### Native PHB1 and PHB2 protein purification

For these assays, we used cultured promastigote forms at the end of the exponential growth. Cells were harvested by centrifugation, the cell pellets were washed three times in Hank’s solution, then resuspended in a phosphate buffer (pH 7.4) containing 0.25 mM sucrose, 1 mM EDTA, 1 mM, DTT, 1% Triton X-100, plus a protease inhibitor cocktail (Complete Mini, Boehringer Mannheim GmbH, Mannheim, Germany). The cell suspension was frozen and thawed three times, then sonicated in a Branson SLP Sonifier (at intervals of 15 s for a total of 2 min, with a 15 s pause). The sonicated fraction was centrifuged at 4000× *g* for 30 min. The extract supernatant was immunochromatographed through a Sepharose GE healthcare column activated with cyanogen bromide 4B (2 ml) previously sensitized with the purified anti-mouse Leish r PHB1 IgGs or rabbit Leish r PHB2, IgGs. The elution was performed at a flow rate of 2 ml/min with a glycine HCl buffer at pH 4.5. The elution product was neutralized and the eluted fraction was dialyzed in 0.1 M ammonium acetate buffer and lyophilized.

### Fluorescence and electron microscopy

For confocal microscopy, *Leishmania* cells in logarithmic growth phase were harvested by centrifugation. The mitochondria were stained by incubation for 30 min at 28 °C in the culture media supplemented with 50 nM Mitotracker Red CMXRos (Invitrogen). After incubation, the cells were rinsed 3 times with PBS for 5 min at 4 °C and fixed with paraformaldehyde at 3.7%, for 15 min at RT. The fixed cells were washed four times with PBS and permeabilized in 10 mM citric acid (100 ml H_2_O; 100 μl NP-40, pH 6) for 5 min at 95 °C. After permeabilization, the promastigote forms were washed three times in PBS and incubated for 30 min in blocking buffer (PBS, pH 7.2; 1% BSA). Subsequently, samples were incubated with anti-Leish r PHB1 or anti-Leish r PHB2, IgGs at a 1/100 dilution in blocking buffer at room temperature for 1 h. Then, for a secondary detection antibody, we used either anti-mouse or anti-rabbit serum labelled with fluorescein isothiocyanate (FITC) (Sigma) diluted in blocking buffer for 1 h at room temperature.

For DNA staining, fixed culture samples were incubated for 15 min in a 10 μg/ml DAPI solution. The stained samples were preserved on slides with a mounting medium (Prolong Antifade Kit, Molecular Probes, Eugene, Oregon, USA) and examined under a Leica DMI6000 confocal laser microscope equipped with a filter system for FITC (mean wavelength 530 nm, maximum 490 nm).

For ultrastructural studies of Leish PHB1 and Leish PHB2 distribution, stationary promastigote forms of *L. major* were centrifuged at 100× *g* for 10 min. The pellet was washed three times with PBS by centrifugation and then fixed with Karnovsky fixative [2.5% v/v glutaraldehyde, 2% v/v formaldehyde in 0.1 M cacodylate buffer, 50 mg CaCl_2_ in100 ml with 0.1 M sucrose (pH 7)] for 14 h at 4 °C. After the washes and dehydration, the samples were embedded in LR White (TAAB, Reading, UK), and sliced into 100-nm-thin sections with ultramicrotome (Leica, Wetzlar, Germany) [17].

Slices were blocked with 0.2 M glycine and either the mouse anti-Leish r PHB1 (1:25) or rabbit anti-Leish r PHB2 was used (1:20) diluted in blocking buffer (PBS 1% bovine serum albumin pH 7.2). As a secondary detection antibody for PHB1, we used an anti-mouse antibodies coupled to 10 nm gold particles (Sigma) diluted 1:125 and incubated for 1 h at 37 °C. For PHB2 detection, we used anti-rabbit antibodies coupled to 25 nm gold particles (1:100).

A negative control was made by incubation with sera from pre-immunized mice or rabbits under the same conditions. Finally, ultrathin sections were stained with uranyl acetate and examined under a LIBRA 120 PLUS Carl Zeiss SMT Transmission Electron Microscope (Oberkochen, Germany ) at 120kV.

### Influence of the Fe^3+^ cation on *L. major* PHB1 and PHB2 expression

The influence of iron, under stress conditions, on the expression of both genes was studied by cultivating promastigote forms for 24 h in RPMI1640 medium plus 10% IFCS, as described above, supplemented with increasing concentrations (0, 5, 10 15, 30, 66, 100, 250 and 500 μM) of ammonium ferric citrate. For the range of ammonium ferric citrate concentrations, we used those described elsewhere [33]. At the end of the cultivation time, the flagellate forms were centrifuged, the total mRNA purified and mRNA levels of the prohibitins were assessed by RT-qPCR.

### Fe^3+^ cation affinity

Fe-NTA agarose was prepared in the affinity column using a commercially available Ni-NTA agarose (Qiagen Inc., Hilden, Germany), in which the Ni ion was replaced by the Fe ion, as described elsewhere [34, 35]. To 750 μl cell lysates, 50 μl Fe-agarose suspension was added and incubated at 4 °C for 6 h in a rotating device, and subsequently washed 3 times in ice-cold PBS and eluted with a solution of 0.02 M sodium acetate, 1 M NaCl, pH 4. The eluted proteins were analysed in 12% SDS-PAGE electrophoresis, transferred to nitrocellulose filters and analysed by Western blot using each of the two anti-PHB antibodies, diluted at a 1:50 ratio, as described above. In parallel the recombinant proteins were electrophoresed as before but the gels were stained in the dark with a solution of potassium ferricyanide III (100 mM K_3_[Fe(CN)_6_]) in 50 mM Tris-HCl; 100 mM NaCl, pH 7.5) for 10 min, and then destain with a solution of 10% trichloroacetic acid/10% in methanol until the bands were completely visible. Albumin was used as a negative control [35].

### Metal catalysed oxidation (MCO) assay

To assess the capability of prohibitins to protect the DNA against oxidative stress, we applied the metal-catalysed oxidation (MCO) methods described by Lim et al. in 1993 [36]. This approach is based on the Fenton reaction, and for the assay, different concentrations of native Leish PHB1 and Leish r PHB2 (125, 250 and 500 μg/ml) were used, mixed with 33 μM FeCl_3_ and 10 mM DTT, for a total volume of 25 μl. Bovine serum albumin at the same concentrations was included as a control. The mixture was incubated for 4 h at 37 °C. After this incubation period, 0.5 μg of DNA from plasmid pRIBOTEX was added and incubated for 4 h at 37 °C. At the end of the incubation period, the integrity of the plasmid DNA was assessed by electrophoresis of 0.8% agarose gels and visualized under UV after staining the gel with SYBR Green.

### Study of *L. major* PHBs under oxidative stress

For the evaluation of *L. major* viability under oxidative stress, a modified MTT [3-(4,5-dimethylthiazol-2-yl)-2,5-diphenyltetrazolium bromide] assay [37] was performed. The MTT conversion from a yellow substrate into a blue crystal product (formazan) measures the total mitochondrial dehydrogenase activity [38]. A total of 2 × 10^6^ parasites in medium RPMI1640 were incubated in a 24-well plate with concentrations of H_2_O_2_ of 0, 10, 25 and 100 nM for 90 min. Multi-well plates were centrifuged at 1500× *g* for 15 min, and the flagellate pellet resuspended in 100 μl of 0.5 mg/ml MTT solution and incubated for 4 h at 25°C. Next, plates were centrifuged at 1500× *g* for 10 min and the pelleted formazan crystals were solubilized in 100 μl of 100% (v/v) dimethyl sulfoxide (DMSO). Finally, the absorbance of each sample was read at 595 nm using a Multiskan® Spectrum microplate reader (Thermo).

Cultures of *L. major* promastigotes were treated for 30 min with 25 and 100 μM H_2_O_2_ fixed (using Karnovsky Fixative; 2.5% v/v glutaraldehyde, 2% v/v formaldehyde in 0.1 M cacodylate buffer, and 50 mg CaCl_2_ in100 ml) and subjected to PHB1 and PHB2 immunolocalization by electron microscopy as described above.

### Statistical analysis

The Tukey-Kramer test of the software package Graph Pad InStat v.3.06 (32 bit) was used to study the significance levels of the different assays, for which *P* < 0.05 (*) was taken to be significant and *P* < 0.001 (***) extremely significant.

## Results

### Cloning, expression and sequence analyses of *L. major* PHBs

**Fig. 1:**
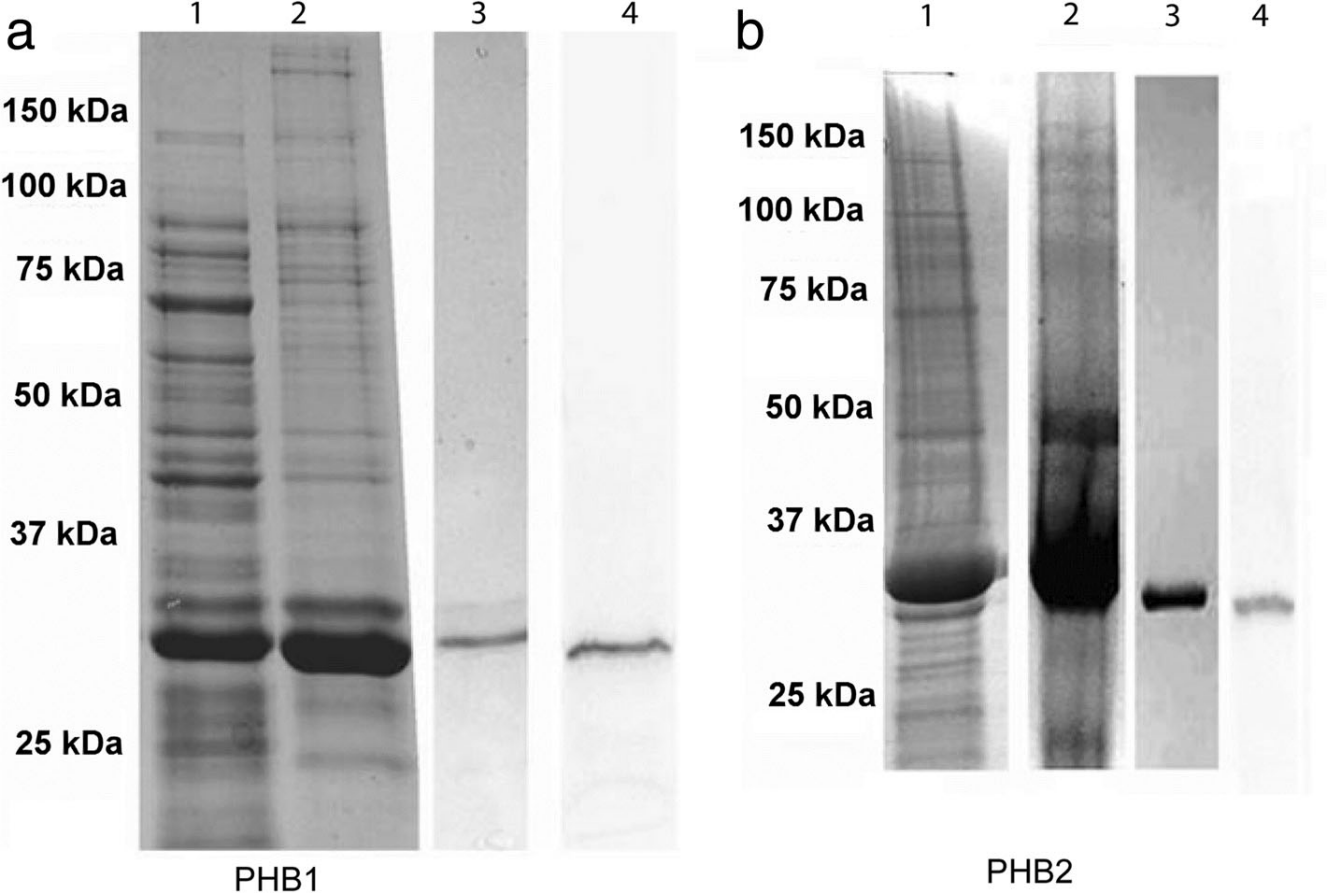
SDS PAGE of *Leishmania* recombinant proteins Leish r PHB1 (**a**) and Leish r PHB2 (**b**). Lane 1: total proteins from the transformed bacteria; Lane 2: protein profile after induction with IPTG; Lane 3: sample after purification with a Ni-NTA column; Lane 4: recognition by Western blot with the antibodies produced against these proteins

The quality of different purified recombinant proteins assayed on SDS-PAGE 12.5% electrophoresis is shown in Fig 1a, b. MALDI-TOF-MS peptide analysis of the 30-kDa band resulted in four fragmentation spectra with a highly significant homology (*P* < 0.05) for the *Leishmania* protein PHB1 XP_001682244.1; (LmjF.16.1610). This protein has a total of 268 amino acids, with a molecular mass of 30,272 Da and an isoelectric point (IP) of 8.42. The conserved PHB domain is present from amino acid 22 to 202 and its topology, either from the hydrophobicity profile or the predictive analysis of the transmembrane regions, shows a hydrophobic region, a signal peptide from aa 1 to 23 and a GPI (glycophosphatidylinositol) binding site located in residue 252. Leish PHB2 appears to be related to PHB1 and, although this gene appears in public databases, it has not been previously characterized. The design of specific primers enabled us to amplify the PHB2 gene from *L. major* genomic DNA. After cloning and the *in vitro* expression of the gene, its identity was confirmed by its MALDI-TOF-MS spectrum.

*Leishmania major* prohibitin 2 (XP_003722404.1; (LmjF.35.0070) had a total of 292 amino acids with a theoretical molecular weight of 32,328 Da and an IP of 9.49. Sequence analysis revealed that Lm PHB2 lacked a signal peptide but had a GPI site at residue 274, and that the conserved PHB domain reached from amino acid 38 to 233.

According to the hydrophilic profile, the two proteins appear to have transmembrane regions with an N-terminal hydrophobic region between amino acids (aa) 8 to 26 in PHB1 and between aa 14 and 33 in PHB2. The two recombinant proteins here obtained have molecular weights around 30 and 32 kDa for Leish r PHB1 and Leish r PHB2, respectively. The molecular mass estimated from the gels shown in Fig. 1a, b coincides with the *in silico* estimations.

The antibodies against Leish r PHB1 recognized a single band of approximately 30 kDa in the lane corresponding to PHB1 (Fig. 1a, Lane 4), whereas the antibodies prepared against Leish r PHB2 showed a single band of only about 32 kDa (Fig. 1b, Lane 4).

**Fig. 2:**
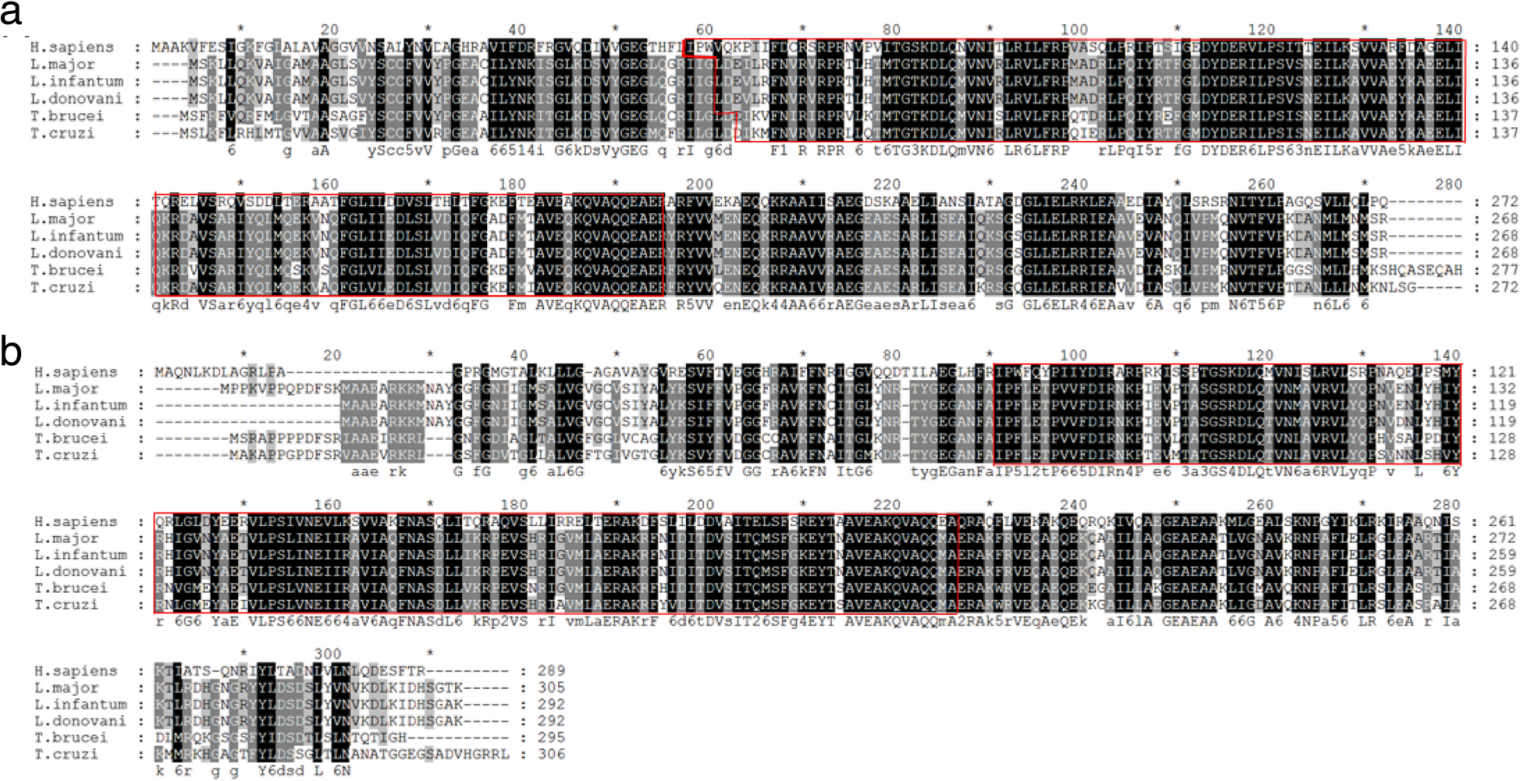
Alignment of PHB1 (**a**) and PHB2 (**b**) of *H. sapiens*, *L. major*, *L. infantum*, *L. donovani*, *T. brucei* and *T. cruzi*. The red boxes indicate the Band 7/SPFH domain superfamily (IPR036013)

The analysis by Clustal Omega of PHB1 and PHB2 of *L. major*, *L. donovani*, *L. infantum*, *T. brucei*, *T. cruzi* and *H. sapiens* is shown in Fig. 2a and b, respectively. The red boxes indicates the Band 7/SPFH domain superfamily (IPR036013).

### Quantification of mRNA expression of *Leishmania* PHBs

**Fig. 3:**
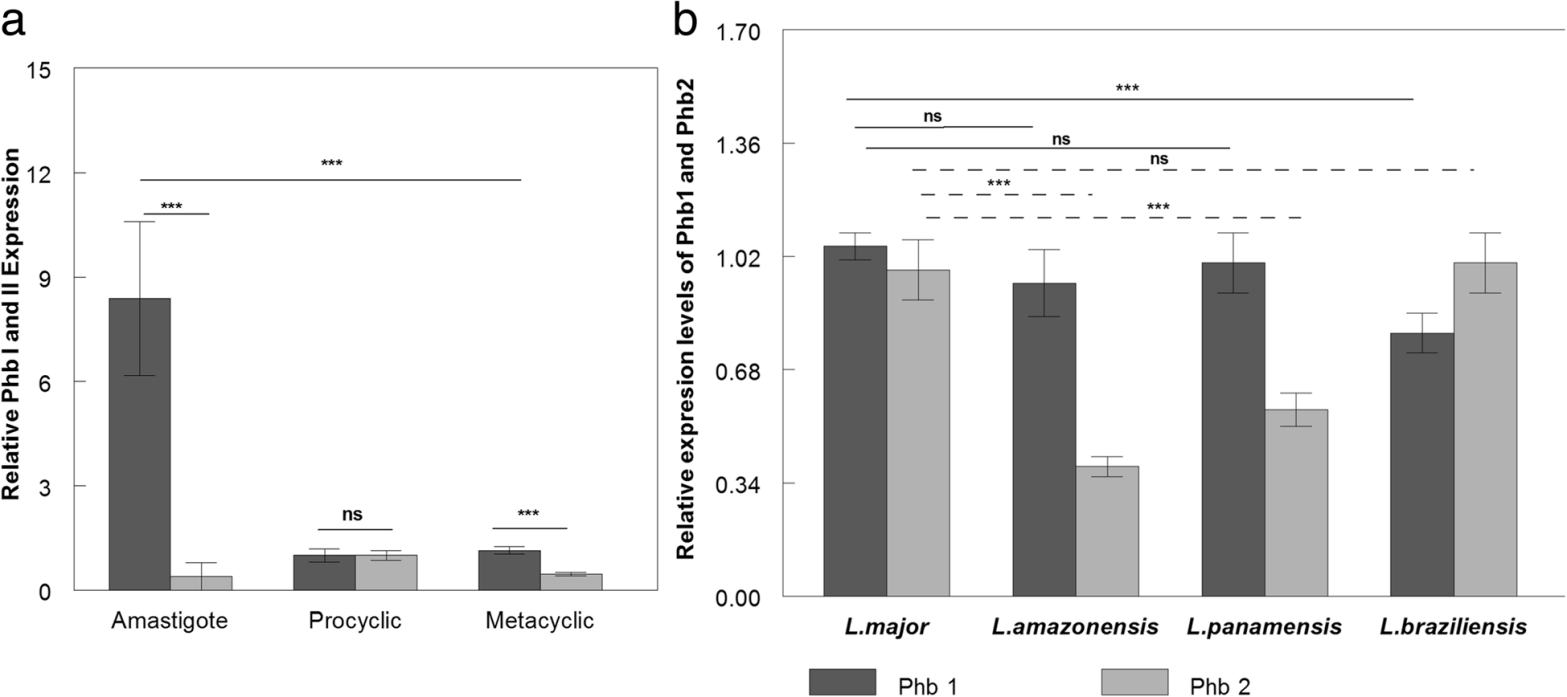
**a** RT-qPCR Leish PHB1 and Leish PHB2 mRNAs in the different developmental forms of *Leishmania major*. **b** Expression levels of Leish PHB1 and Leish PHB2 mRNAs in the promastigote forms of different *Leishmania* species. Dark-grey columns correspond to PHB1, and light-grey to PHB2. The level *** *P* < 0.001 is considered extremely significant; ns: non-significant (analysed by the Tukey-Kramer multiple comparisons statistical test).The bars in the graphs represent the mean ± the standard deviation (SD)

The Fig. 3a shows the relative mRNA expression levels of PHB1 and PHB2 in the different developmental forms of *L. major*. The mRNA levels found for PHB1 were significantly higher than those for PHB2 in amastigote forms. The promastigote forms registered similar expression, whereas in metacyclic infective promastigote forms, although the expression levels were low, PHB1 showed a higher expression than PHB2. The expression level ratio PHB1:PHB2 was 22.02 for amastigotes, 1 for promastigotes and 2.50 for metacyclic promastigotes.

The Fig. 3b shows the results of the RT-qPCR experiments to determine the relative expression levels of PHB1 and PHB2 mRNAs in promastigote forms from different *Leishmania* species. We found no significant differences between the expression levels for PHB1 in *L. major*, *L. amazonensis* or *L. panamensis*, while the PHB1 expression levels in *L. braziliensis* were significantly lower, with a higher expression of PHB2. The ratio between the PHB1:PHB2 expression was 1.07 for *L. major*, 2.41 for *L. amazonensis*, 1.78 for *L. panamensis* and 0.79 for *L. braziliensis*.

### PHB1 and PHB2 localization in *L. major* promastigote forms

**Fig. 4:**
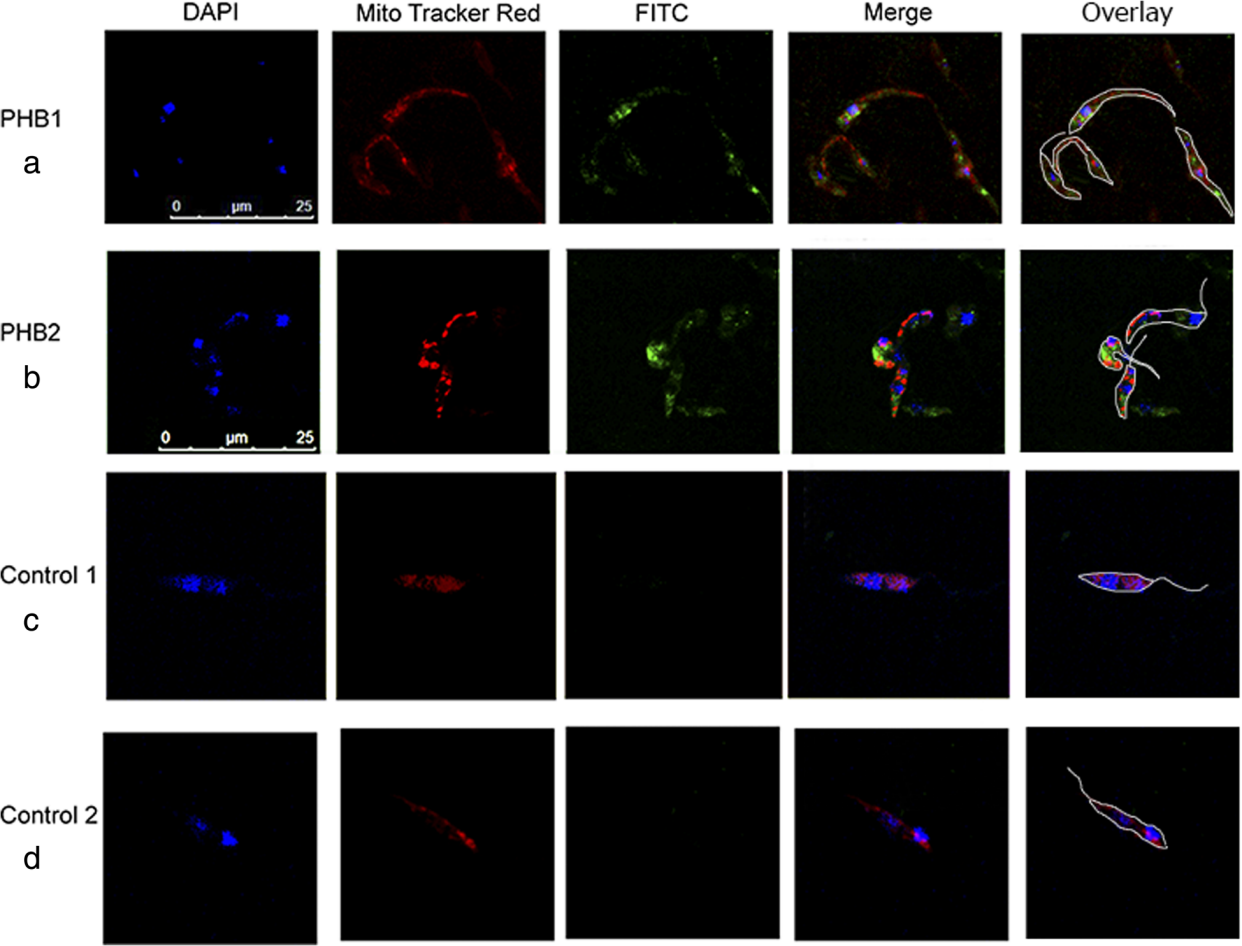
Confocal laser microscopy of *L. major* promastigote cells probed with anti-Leish r PHB1 (**a**) and anti-Leish r PHB2 (**b**). The mitochondria were stained in red with MitoTracker. The DNA (nucleus and kinetoplast) is clearly visible stained in DAPI blue. PHB1 fluorescence scatters throughout membranes. PHB2 is distributed throughout the cell mitochondria. In some of the promastigotes, fluorescence is visible in the nuclear region. The control 1 samples (**c**) were treated with pre-immune sera and the control 2 samples (**d**) were treated only with the secondary antibody. *Scale-bars*: 25 μm

The results of confocal laser microscopy studies using anti-Leish r PHB1 and anti-Leish r PHB2 antibodies in *L. major* promastigotes are shown in Fig. 4. The mitochondria are stained in red with MitoTracker, PHBs in green when recognized by the specific antibodies labelled with FITC, and the nuclei and the kinetoplast in blue with DAPI (4',6-diamidino-2-phenylindole). Control 1 are samples treated with pre-immune sera whereas Control 2 consists of samples treated with the secondary antibody only.

PHB1 appears distributed throughout the cell membranes and no fluorescence was detected in the nuclei. In the case of PHB2, it is distributed in the mitochondria with some fluorescence visible in the nucleus.

**Fig. 5:**
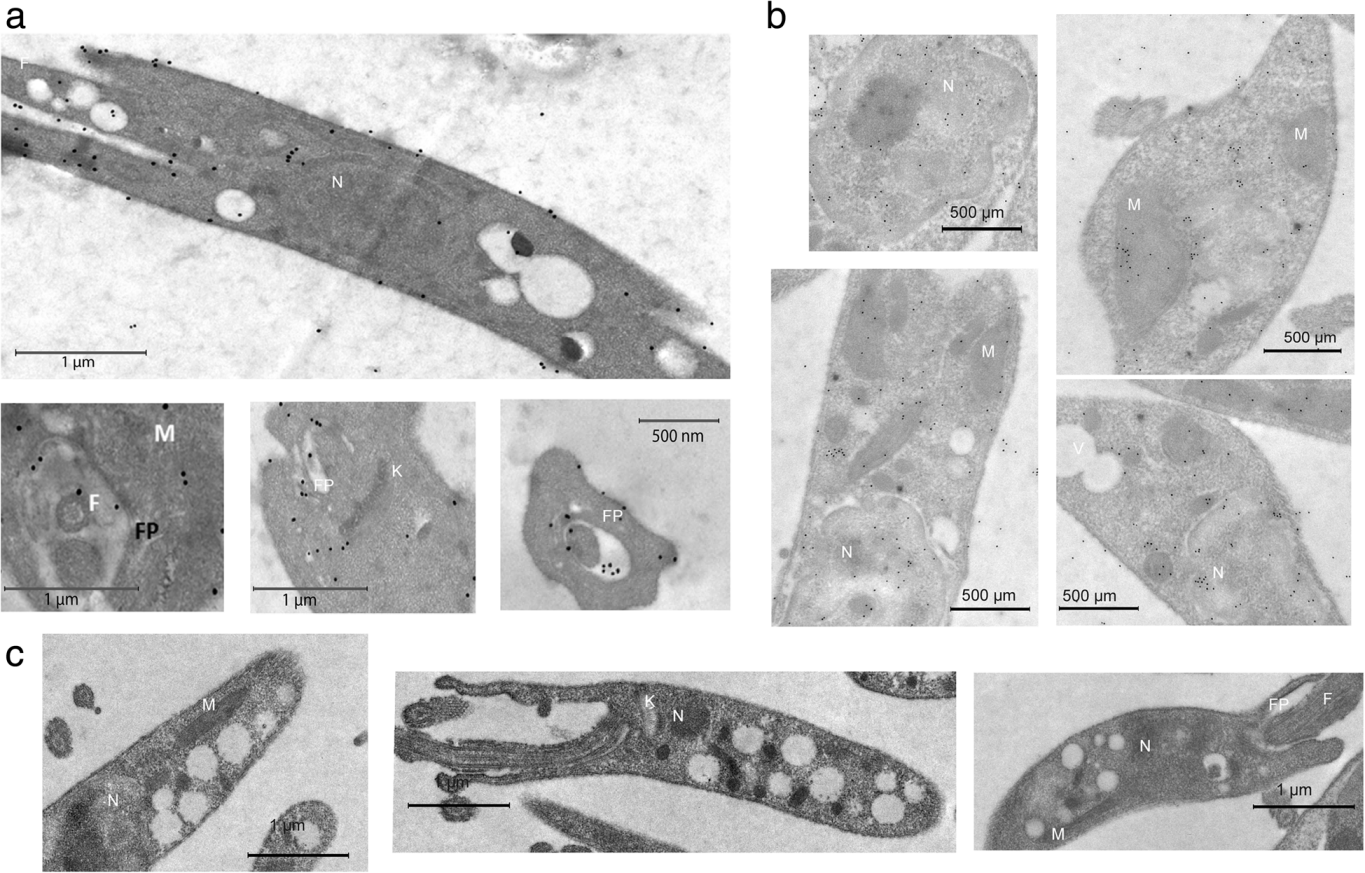
Immunolocalization of PHBs in *L. major* promastigotes. **a** PHB1 antibodies labelled with gold particles located in the cytoplasmic membrane, close to the mitochondria (M); in the vacuole membranes (V); in the kinetoplast (K); in the flagellar pocket (FP). **b** PHB2 antibodies recognizing antigens in the nucleus (N) and mitochondria (M). Isolated gold particles visible in the vacuole (V) and in the flagella (F). **c** Control cells treated with pre-immune sera

Ultrastructural immunolocalization studies using anti-Leish r PHB1 IgGs, showed gold marks in the plasma membrane, close to the mitochondrial membranes, in vacuoles, the kinetoplast and flagellar pocket (Fig. 5a).

The anti-Leish r PHB2 IgGs antibodies recognized antigens clustered in the chromatin, and in the vicinity of the nuclear membrane. Groups of gold particles were also visible in the cytoplasm near the mitochondria (Fig. 5b). The control samples treated with pre-immune sera are shown in Fig. 5c.

### The effect of iron cations

When comparing the amino acid sequences of the two *Leishmania* prohibitins, the present study revealed that both contained putative motives for interaction with iron. These domains were similar to those found in certain bacteria and in the ferritin molecular region that interacts with this metal [39]. According to previous studies, PHBs have the ability to associate with certain metals, including iron. The sequence of proteins that interact with iron contain multiple E-x-x-E or R/K-E/D-x-x-E or R/K-E-x-x-E/D motives, and those that bind to iron have the motive E-D-x-D-E [40].

**Fig. 6:**
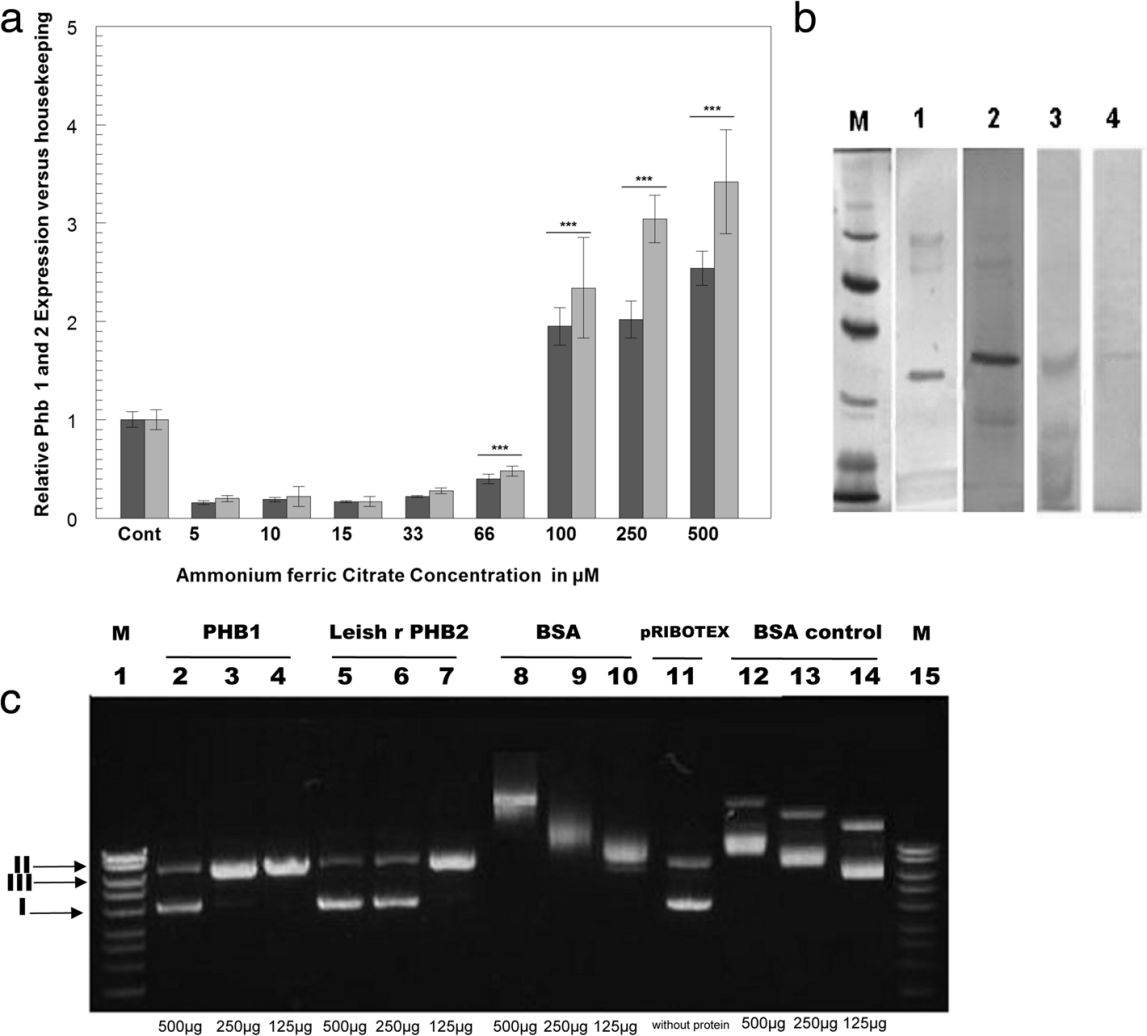
**a** Expression levels by RT-qPCR of PHB1 and PHB2 in media supplemented with different concentrations of ammonium ferric citrate, measured by RT-qPCR. The dark-grey columns correspond to the PHB1 expression levels. The light-grey columns correspond to the PHB2 expression levels. The bars in the graphs represent the mean ± the standard deviation (SD). **b** Purification of PHB1 and PHB 2 from *L. major* homogenate by affinity chromatography using a Fe-NTA column. Identification by Western blot using anti-Leish r PHB1 (Lane 1) and Leish r PHB2 antibodies (Lane 2). Lane 3: native PHB1 eluted from the Fe-NTA column and stained with K_3_[Fe(CN)_6_III]and (4) Leish r PHB2 also stained with K_3_[Fe(CN)_6_III]. 2. *** *P* < 0.001 by the Tukey-Kramer multiple comparisons statistical test. **c** Protective effect of PHB1 and PHB2 against DNA damage caused by hydroxyl radicals. The positions to which supercoiled (I), relaxed circular (II) and linear (III) monomeric DNAs migrated are indicated on the left of the picture. Lanes 2, 3 and 4: native PHB1 at 500, 250 and 125 μg/ml, respectively; Lanes 5, 6 and 7: recombinant Leish r PHB2 at 500, 250 and 125 μg/ml, respectively; Lanes 8, 9 and 10: controls with BSA at 500, 250, and 125 μg/ml, respectively; Lane 11: control plasmid pRIBOTEX DNA; Lanes 12, 13 and 14: BSA incubated with the probate DNA, but without DTT in the solution (not exposed to hydroxyl radicals); Lanes 1 and 15: reference Hyperladder II

Starting at aa 107, PHB1 contained the sequence GL**D**Y**DE**RILP, while PHB2 had the sequence RV**E**QA**E**Q**E**K from aa 211 onwards. In bold and underlined constitute in the protein sequence, the amino acids in which the metal would bind. These observations led us to explore the effect of iron on the mRNA expression of *Leishmania* PHBs. Figure 6a shows the PHB mRNA expression levels in the presence of increasing amounts of ammonium ferric citrate ranging between 100–500 μM. The ΔCt expression of Leish PHB1 relative to the normal culture medium (control) was 1.95 ± 0.19 and 2.54 ± 0.17 times. In the case of PHB2, the expression levels in the presence of ammonium ferric citrate were 2.34 ± 0.51 and 3.42 ± 0.53 higher than the control at 100 and 500 μM, respectively. Starting at a concentration of 66 μM ammonium ferric citrate, the expression levels of PHB2 were significantly higher than those of PHB1.

The capacity of *L. major* PHBs to bind Fe ions was also examined using affinity chromatography and SDS electrophoresis, followed by specific staining of the Fe cation. When the total proteins from a homogenate of *Leishmania* promastigotes were purified for their capacity to bind to the matrix of the Fe-NTA column, the antibodies against the recombinant prohibitins (Leish r PHB1 and Leish r PHB2) were able to recognize bands of 30 and 32 kDa (Fig. 6b, Lanes 1 and 2, respectively). Similar bands were recognized when the gels were stained with K_3_[Fe(CN)_6_] (Fig. 6b Lanes 3 and 4, respectively).

### Study of the role of *Leishmania* PHBs under oxidative stress

The protective role of prohibitins against oxidative stress has been supported by experimental evidence in various cell types [41, 42]. Here, we tested the capacity of prohibitins to prevent DNA damage in an oxidative medium linked to Fe III ions and, thereby blocking the Fenton reaction. The Fenton reaction is a modified Haber-Weiss reaction driven by Fe^3+^ [43]. In addition to iron in oxidation state (III), dithiothreitol (DTT) was used as an electron donor.

After incubations of *Leishmania* PHBs (PHB1 native and Leish r PHB2) with plasmid pRIBOTEX DNA and DTT in order to generate the Fenton and Haber-Weiss reaction, samples were run in agarose gel at 0.8%. Figure 6c shows that native PHB1 at a concentration of 500 μg/ml protected pRIBOTEX DNA against the nicking caused by hydroxyl radicals, whereas the recombinant PHB2 protein was more efficient, achieving this protection down to a 250 μg/ml concentration. When we compared these results to the DNA incubated with BSA, despite the mobility change caused by this protein (Fig. 6c, Lanes 8–10) [44], we observed a more pronounced degradation when compared to the control (Lanes 12–14). Therefore, even when a nicking activity was detected at PHB1 concentrations of 250 and 125 μg/ml, and PHB2 at 125 μg/ml, the presence of *Leishmania* PHBs slowed down the process of DNA damage.

### Ultrastructural localization of *Leishmania* prohibitins under H_2_O_2_ stress

As described above, under normal conditions in promastigotes cells, PHB1 was found in the cytoplasmic membranes near the mitochondrial membranes (M), in vacuolar membranes (V), the kinetoplast (K) and the flagellar pocket (FP) (Fig. 5a). Once promastigotes cells were treated with 25 and 100 μM H_2_O_2,_ the labels were detected mainly in the flagellar pocket, in the membranes of the flagella, and some were scattered in the axoneme as well as in the nucleus. The images also show light electron-dense membrane evaginations or protrusions measuring roughly 100 nm, suggesting the presence of exovesicles (exosomes and ectosomes).

**Fig. 7:**
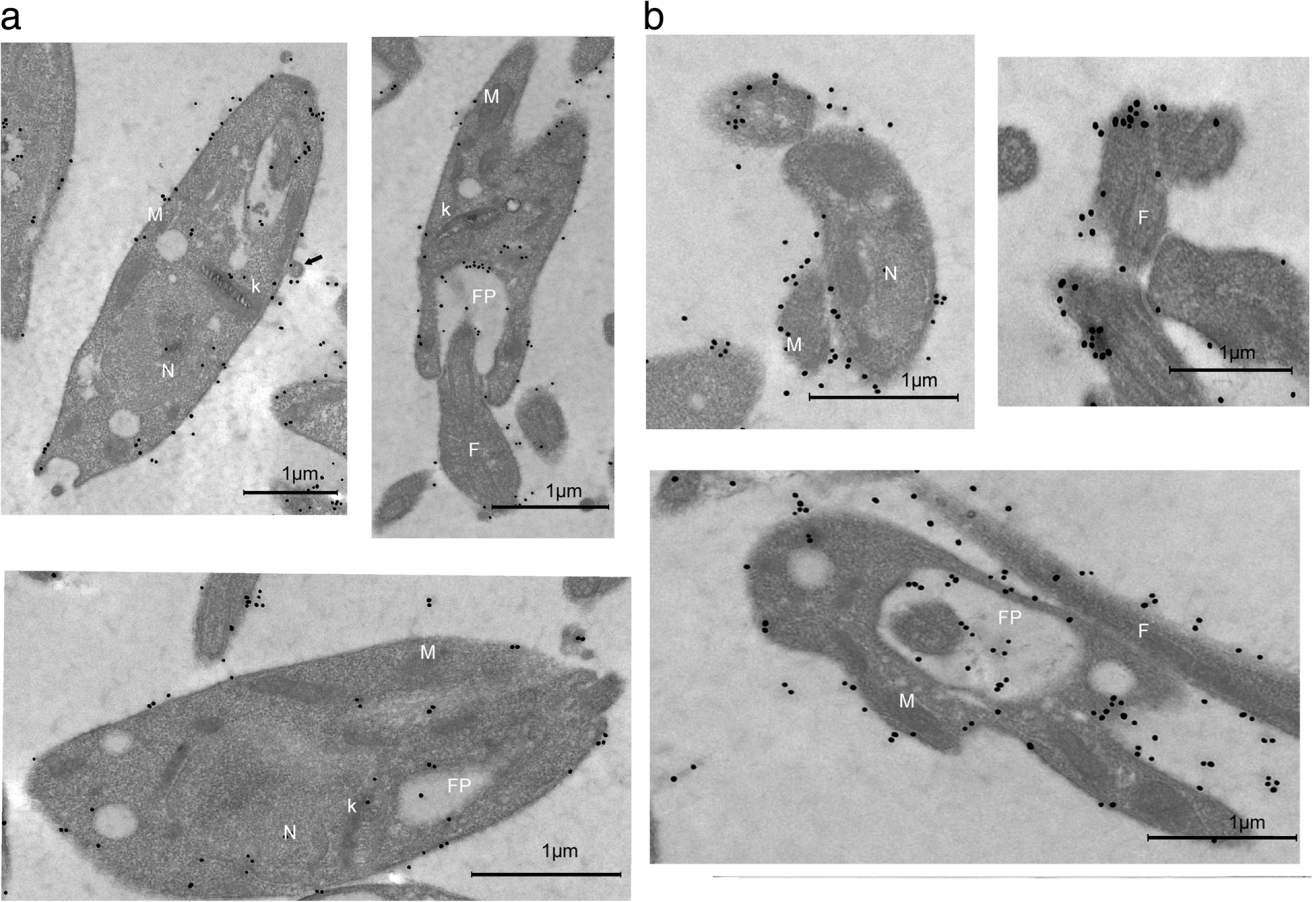
Electron immunocytochemical assay to determine the location of PHB1 in *L. major* promastigote forms after treatment with H_2_O_2_. **a** In cells treated with 25 mM H_2_O_2_ gold particles are visible in the flagellar pocket (FP), nucleus (N) and some in the kinetoplast membrane. The micrograph shows membrane evaginations similar to ectosomes. Arrows point to groups of gold labels associated with free exovesicles of approximately 100 nm at the periphery of the parasite. **b** In cells treated with 100 μM of H_2_O_2_ the gold labels are visible in the membrane, the flagellar pocket, and the flagellar membrane

In the case of PHB1 *Leishmania* cells treated with H_2_O_2_, a 2.37–2.6-fold increase was found in the number of gold grains per micron or square micron in the cytoplasmic membranes and in the flagellar pocket matrix, respectively. Additionally, we detected an increment of the PHB1 released through the flagellar pocket, due perhaps to an increase of the secretome. This active secretion of PHB1 contributed to the dispersion of PHB1 observed in individuals subjected to oxidative stress (Fig. 7a, b).

**Fig. 8:**
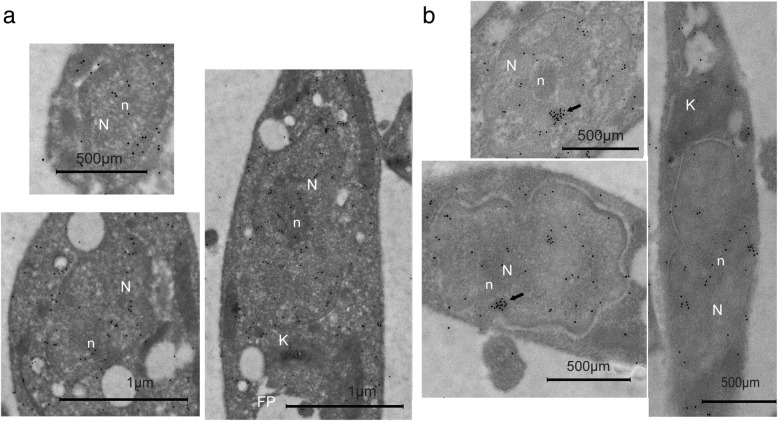
Electron immunocytochemical studies to follow the location of PBH2 in *Leishmania major* promastigote forms after treatment with H_2_O_2_. **a** In cells treated with 25 mM H_2_O_2_ the gold labels were observed inside the nucleus (N) in clusters close to the nucleolus. Some dispersed gold particles appear also near the kinetoplast (K) and around the flagellar pocket (FP), or around the vacuoles. The arrows point at clusters of gold marks in the nucleus near the nucleolus. **b** In cells incubated with 100 mM H_2_O_2_, some marks appear in the nucleus (N) but less than in the previous treatment. The kinetoplast membrane has a group of marks (arrow) (K), and some gold labels are clustered outside the protozoa

In the case of PHB2 after the same treatment (Fig. 8b), gold marks were visible in the nucleus, near the nucleolus (Fig. 8a) as well as scattered forms near the mitochondria and the kinetoplast membrane.

## Discussion

Prohibitins form a highly conserved protein group with a high degree of sequence homology among species [45, 46]. So far, the exact biological function of PHB1 is not clear and it shares a 50% similarity with another member of the family, namely PHB2.

*Leshmania major* PHB1 has a 43% sequence similarity and a similar size to its human counterpart, i.e. 272 aa (molecular mass of 29,840 Da), and in addition it has four sites for putative motives of phosphorylation for kinases [47]. Regarding PHB2, its function in trypanosomatids has not been described to date but it shows a 46% identity with the human PHB2 and has an N-terminal transmembrane region between residues 14 and 33.

Although PHB1 and PHB2 have no evident mitochondrial targeting signal [48], it is known that no such signal has been identified in some trypanosomatid mitochondrial proteins, such as TcP5CDH [49] and the large repertoire of MCPs.

In early PHB studies based on observations in cancer cells [9], their location was thought to be restricted to the mitochondria. However, recent studies in endothelial and *L. donovani* cells have shown that prohibitins appear in the plasma membrane as well [13, 50].

In mammalian cells, the loss of prohibitins has serious consequences for mitochondrial integrity, causing greater fragmentation of these organelles. Additionally, the lack of prohibitins leads to a cytochrome C release that triggers cell apoptosis, and the loss of the F0 subunit of the ATP-synthetase, which triggers proton release and ultimately cell death [5, 51, 52]. Hence, prohibitins are essential components in the machinery for mitochondrial function [53].

Our experiments with confocal laser microscopy showed that PHB1 is located in the membrane and in the vicinity of the mitochondria (Fig. 4), and these locations were confirmed in our EM Immunolocalization experiments. On the other hand, for PHB2 we detected nuclear clusters close to the nuclear membrane and as dispersed marks inside the mitochondria. PHB2 is located exclusively inside the parasite cell, whereas PHB1 also had isolated marks outside the cytoplasmic membrane, associated with extracellular vesicles of the secretome together with other proteins such as actin and HP70 (results not shown).

Under normal conditions, PHB2 passes from the mitochondria to the nucleus, taking part in a DNA-stabilization mechanism [51]. Recently, it has been reported that inhibition of PHB2 arrests cell proliferation by blocking the cell cycle and suppressing DNA synthesis. This inhibition changes the nuclear morphology, altering its functionality [54]. These types of results suggest that the nuclear location of PHB2 in *L. majo*r is related to stabilization mechanisms of the parasite nuclear components.

In 2010, Týc et al*.* [12] demonstrated that PHB1-deficient *T. brucei* developed mitochondrial alterations, without affecting the kinetoplast, but causing a delay in the division time, thus implying that this protein also forms part of the machinery necessary to complete the parasite cell cycle.

Our results, after studying mRNA expression levels in the different phases of the biological cycle of *Leishmania*, revealed that the highest PHB1 mRNA expression levels occurred in amastigotes and in metacyclic forms, thus corroborating the findings of Jain et al. [13] for *L. donovani* (Fig. 2a).

It is surprising that this high level of PHB1 expression occurs in intracellular amastigotes, where mitochondrial size is reduced. Nevertheless, inside the host cell amastigotes are highly active in cell division. It is tempting, therefore, to speculate that increased PHB1 expression could be related to its chaperone activity by helping the reprocessing of the parasite mitochondrial structure and morphogenesis. The significant differences found in mRNA levels likely correlates with the protein levels for the different forms studied here, and reveals that *Leishmania* has strong transcriptional regulation for PHB genes.

The findings that PHB1 was also localized on the parasite surface and has greater expression in metacyclic infective promastigotes, as well as in amastigotes, suggest that PHB1 appears on the surface to protect the parasite against the ROS generated by the host-cell phagosome. This role appears to be similar to that reported in cultured cells where PHB1 protects against oxidative stress [40, 55]. Conversely, PHB2 mRNA expression levels are reduced in amastigotes, and in the infective metacyclic forms. Our analysis of the expression-level ratio of PHB1 to PHB2 in promastigotes from different species (Fig. 3b) revealed that the most virulent *L. major* strain exhibited a PHB1:PHB2 ratio close to 1. This finding implies that PHB expression levels could be related to the greater capacity of multiplication and survival of the intracellular amastigote forms of the *L. major* strains, which is the most virulent of the strains studied here.

Studies in liver cells have found a dose-dependent correlation between the Fe^3+^ ion and the production of PHBs [35, 40]. *Leishmania* prohibitins have domains for iron affinity, and our results confirmed that prohibitin mRNA expression levels depend on the iron dosage, especially in the case of Leish PHB2 (Fig. 5a). This correlation supports the idea that prohibitins are involved in the iron homeostasis of the cell. Likewise, in human cells, the presence of phosphorylation sites in the *Leishmania* iron-binding domain suggests that the modification of these sites may be related to cell-signalling processes. In particular, the phosphorylation of tyrosine residues within this domain may affect its capacity to bind iron, thus regulating the intracellular traffic of the ion.

The function of PHBs against oxidative stress has been demonstrated in higher eukaryotic cells [40, 55, 56]. Similarly, our results show that *Leishmania* PHBs protect the DNA from the breakage caused by reactive oxygen species (Fig. 6c). When *Leishmania* cells were treated with different concentrations of hydrogen peroxide, PHB1 migrated towards the plasma membrane, the flagellum’s paraxial rod and in extracellular vesicles (Fig. 7a, b). The increase in the PHB1 release through the flagellar pocket in the protozoon treated with the oxidative stress suggests an increase in the secretome of the parasites. In the same experiments, PHB2 shifted towards the nucleus and to vicinity of the kinetoplast (Fig. 8a, b). We deduce that the presence of PHB2 in the nucleus under normal conditions could be involved in counteracting the action of the free radicals mentioned above, thus preserving the integrity and functionality of the DNA. Therefore, PHBs help to eliminate the oxidative stress, inducing the establishment and multiplication of the parasite in this hostile environment and boosting its infectivity. Finally, the differences in protein sequence between *Leismania* and human PHBs, in agreement with Dias et al. [14] could potentially be exploited in vaccination and drug-design strategies.

## Conclusions

In the present study, we demonstrate a correlation between PHBs mRNA expression levels and the presence of Fe^3+^ ions. The mRNA expression was higher for PHB1 in amastigote forms than in the other forms. We conclude that the capacity of prohibitins to prevent DNA damage in an oxidative medium is linked to the affinity for Fe^3+^ ions, thereby blocking the Fenton reaction. PHB1 and PHB2 proteins changed their cellular location in the presence of H_2_O_2_, and thus we propose that PHBs exert a protective effect on membranes and DNA against ROS. This effect enhances the survival capacity of the protozoan by controlling the ROS from the phagosome of the macrophages where the parasite multiplies. In agreement with the proposal made by Dias et al [14], we conjecture that a blockage of PHB action, either by immunological methods or by drugs, could help in the fight against this disease that affects millions of people around the world.

## Abbreviations

BSA: Bovine serum albumin
DAPI: (4′,6-diamidino-2-phenylindole)
DTT: Dithiothreitol
FP: Flagellar pocket
GPI: Glycophosphatidylinositol
IP: Isoelectric point
K: Kinetoplast
Leish r PHB1: *Leishmania major* recombinant prohibitin 1
Leish r PHB2: *Leishmania major* recombinant prohibitin 2
M: Mitochondrial membranes
PBS: Phosphate-buffered saline
PBST: PBS + Tween20
PHB1: Prohibitin 1
PHB2: Prohibitin 2
PHBs: Prohibitins
Rb: Retinoblastoma
ROS: reactive oxygen species
SPFH: Stomatin-prohibitin flotillin-HflC/K
V: Vacuolar membranes
